# Transparency of clinical trials in pancreatic cancer: An analysis of availability of trial results from the ClinicalTrials.gov database

**DOI:** 10.3389/fonc.2022.1026268

**Published:** 2023-01-06

**Authors:** Ren-Qian Huang, You Zhou, Hai-Xia Zheng, Dan Wang, Xiao-Yi Zheng, Zhao-Shen Li, Liang-Hao Hu

**Affiliations:** ^1^ Postgraduate training base in Shanghai Gongli Hospital, Ningxia Medical University, Shanghai, China; ^2^ Department of Gastroenterology, First Affiliated Hospital of Naval Medical University, Shanghai, China; ^3^ Pancreatic Center, Department of Gastroenterology, Affiliated Hospital of Yangzhou University, Yangzhou University, Yangzhou, Jiangsu, China

**Keywords:** transparency, Clinicaltrials.gov, clinical trials, publication of results, pancreatic cancer

## Abstract

**Background:**

Pancreatic cancer (PC) is a highly malignant tumor of the digestive system. As clinical trials involving PC are increasingly being conducted, the transparency of the generated data has become an important issue of concern. In other areas of medicine, clinical trial transparency presents a worrying state of affairs. However, at present, there has been no study examining the transparency of data derived from PC clinical trials.

**Methods:**

A comprehensive search was conducted in the ClinicalTrial.gov database for clinical trials investigating pancreatic cancer as of June 2022. We examined the availability of clinical trial results and recorded the characteristics of the trials.

**Results:**

A total of 856 trials were included in this study, of which 668 were completed and 188 were terminated or suspended. The results of 626 trials (73.13%) were available, of these 230 trials (26.87%) did not disclose any information on the trial data in any form. The publication rate for trials with available results was 86.10%, but the report rate on ClinicalTrial.gov was only 39.78%.

**Conclusion:**

Although approximately 90% of clinical trial investigating interventions on patients with PC have published study results, 30% of trials did not report any findings, and the disclosure of trial results from ClinicalTrial.gov was unsatisfactory. In general, there is still room for improvement in the transparency of PC clinical trials.

## 1 Introduction

The lack of transparency in clinical trials is a long-standing concern that has attracted much attention (1). In 1997, the National Institutes of Health (NIH) and the Food and Drug Administration (FDA) collaborated to develop ClinicalTrials.gov, which was later promoted worldwide and finally made available to the public in 2000 (2). ClinicalTrials.gov was designed to provide clinicians worldwide with detailed information about clinical trials on a variety of diseases. Initially, clinical trials were required to be registered within 21 days of enrolling the first subject and the submission of the trial results was not mandatory (3). However, the results of some clinical trials (e.g., trials not meeting the primary endpoint) and adverse events may have been selectively disclosed due to the interests of the study sponsor, leading to undesirable consequences such as limiting knowledge dissemination and understanding of the clinical benefits and harms to patients (4). Therefore, to address this issue, Section 801 of the US Food and Drug Administration Amendments Act (FDAAA) of 2007 expanded the legal requirements for trials on ClinicalTrials.gov, in which trials were required to report results within one year after completion, regardless of whether the trial results had been published (5). However, several studies have found that most researchers ignore this provision. In a transparency study in clinical gastroenterology trials, results were available for 1824 of a total of 2429 clinical trials, but only 29% were disclosed on ClinicalTrials.gov (6). In another study on the transparency of clinical trials of gastrointestinal endoscopy studies, results were available for 751 of 923 trials and only 22% were reported on ClinicalTrials.gov (7).

Pancreatic cancer (PC) is a highly malignant tumor of the digestive system and is one of the leading causes of death among cancer patients worldwide (8, 9). The prognosis for PC is poor, with overall survival rates of 24% and 9% at 1 and 5 years, respectively (10). In the United States, PC ranks fourth in malignancy-related deaths, while in China, PC ranks sixth (11, 12). In recent years, the diagnosis and treatment of PC have been further improved with the continuous development of endoscopic techniques, imaging, and pathology, as well as advances in antitumor drugs, radiotherapy techniques, and surgical concepts (13–16). In this process, a large number of clinical trials on PC have been conducted in countries around the world. The disclosure of clinical trial results, whether good or bad, is critical for researchers, clinicians, and patients. It can help researchers design research schemes and assist clinicians in formulating better treatment schemes for patients. However, in the field of PC, there is still a lack of relevant studies on the transparency of clinical trials.

Therefore, the objective of this study was to assess the availability of clinical trials results related to studies on PC registered on the ClinicalTrials.gov database.

## 2 Methods

### 2.1 Search strategy and data sources

Based on the advanced search function of ClinicalTrials.gov, we used the search terms “pancreatic tumor” and “pancreatic cancer” on 1 June 2022 to retrieve eligible trials. We did not set restrictions on the search terms to include as many clinical trials as possible.

### 2.2 Eligibility criteria

Clinical trials were enrolled in this study if they met the following inclusion criteria (1): all registered adult clinical trials showing completion, suspension, or termination as of 1 June 2020 (Referring to previous studies, a two-year follow-up period was allowed to disclose the results of clinical trials (7)) (2); clinical trials on PC.

The exclusion criteria were as follows (1): non-PC clinical trials (2); clinical trials involving children (3); clinical trials showing other statuses except for completion, suspension, and termination (4); clinical trials without specific completion time.

### 2.3 Data extraction and transparency of studies

Based on the information provided by ClinicalTrials.gov, such as the NCT number, trial title, trial purpose, intervention method, study site and researcher, three authors (RQH, YZ, and HXZ) searched the PubMed, Google Scholar, and Web of Science database for trial results published in peer-reviewed journals. Furthermore, based on the reference links in ClinicalTrials.gov, we also considered that the trial results were published if the contents of publication were consistent with the contents of the trial registration. Any disagreements were resolved through the authors’ consultation.

The following characteristics of clinical trials were recorded: availability of results, registration date, duration of trials, type of study, study status, type of intervention, phase of study, source of funding, type of endpoints, whether or not the primary endpoint was met, number of study sites, country of origin and reasons for suspension and termination of trials.

### 2.4 Statistics

First, we divided all included trials into two groups based on the availability of the trial results. Univariate analysis was used to compare significant differences in trial characteristics between the two groups. Multivariate logistic regression analysis was used to identify characteristics that were independently associated with the availability of the results of the clinical trial. The effect sizes were expressed as odds ratios (OR) with 95% confidence intervals (CIs). Furthermore, trials with available results, were divided into two groups based on whether they met the primary endpoint and were then compared the disclosure rate of results on ClinicalTrials.gov and the publication rate in peer-reviewed journals between the two groups. All statistical analyses were performed using SPSS 26.0 software. Categorical variables were expressed using frequencies and proportions and analyzed using Pearson’s χ^2^ test. Continuous variables were expressed using the median and interquartile range and analyzed using the nonparametric Mann-Whitney test. A *P*-value <0.05 was considered statistically significant.

## 3 Results

### 3.1 Search results

**Figure 1:**
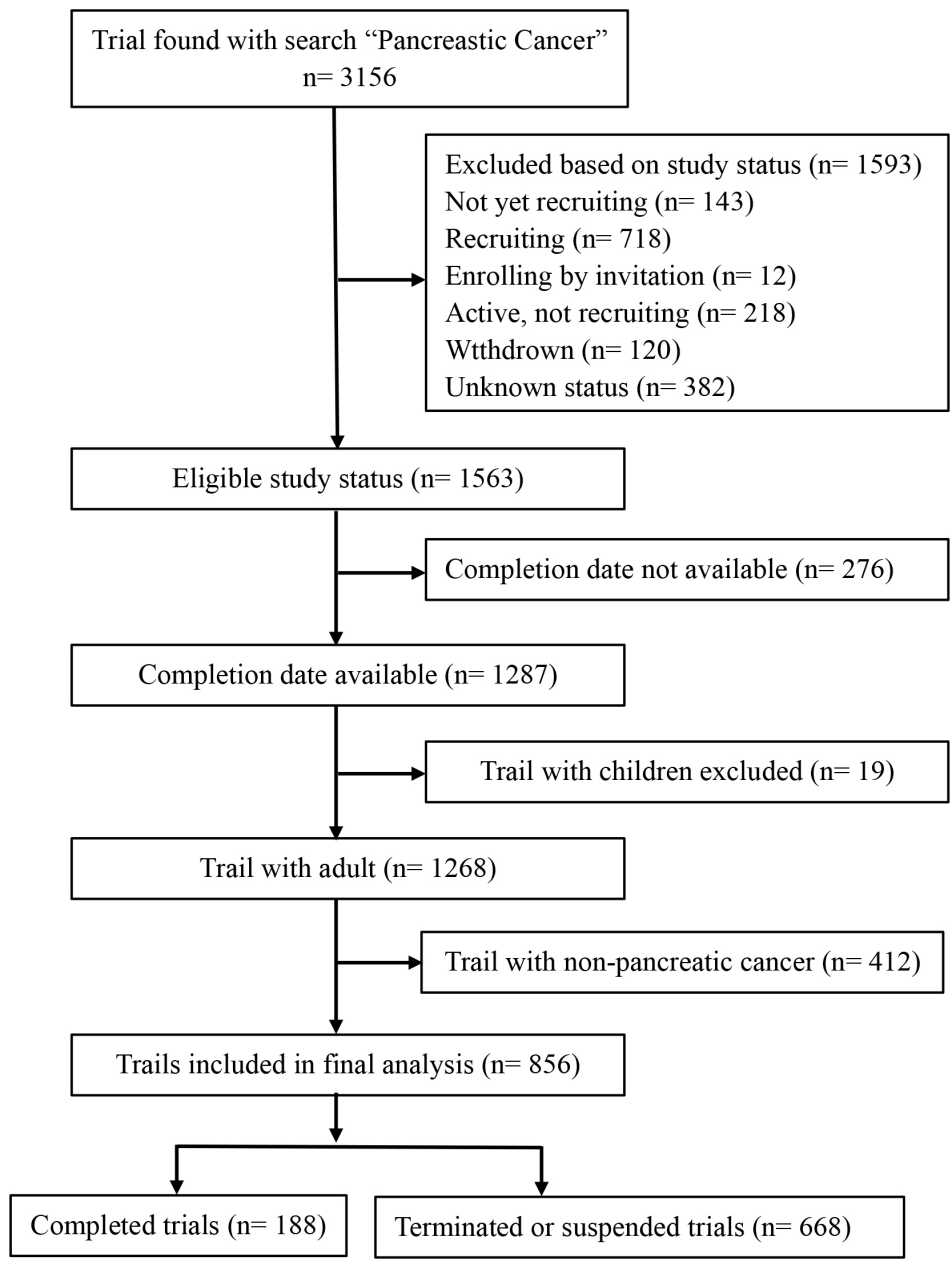
Flow diagram of clinical trials selection..

**Figure 1** shows the selection process for clinical trials. A total of 3156 trials related to PC were extracted from ClinicalTrials.gov during the initial search, of which 1593 were excluded because of study status, 276 were excluded due to the date of completion, 19 were excluded for including children, and 412 were excluded because they were not trials involving only pancreatic malignancies. Ultimately, 668 completed trials and 188 terminated or suspended trials were used for statistical analysis.

### 3.2 Trial characteristics

**Table 1** presents the characteristics of the included clinical trials. Of a total of 856 trials, study results were available for 626 trials, of which 249 (249/626, 39.78%) had results disclosed on ClinicalTrials.gov, 539 (539/626, 86.10%) had results published in peer reviewed journals, and 164 (164/626, 26.20%) were both disclosed on ClinicalTrials.gov and published in peer-reviewed journals. Of the 626 trials with available results, 541 (86.42%) met the primary endpoint. The results of these studies were presented most often in the form of publications (*P<*0.001) and studies not meeting the primary endpoint rarely disclosed the results on ClinicalTrials.gov (*P<*0.001). Interventional trials (578/757, 76.35%) were more likely to have available results than observational trials (48/99, 48.48%) (*P<*0.001). Compared to suspended or terminated trials (112/188, 59.57%), completed trials (514/668, 76.95%) were more likely to report results (*P<*0.001). In comparison, intervention-type trials associated with chemotherapeutic drugs and biological therapies were more likely to have results (*P<*0.001). Phase II studies and the above trials were more likely to report results than trials with unknown phase status or Phase I trials (*P<*0.001). Trials with clinical endpoints (525/683, 76.87%) were more likely to disclose results than trials with nonclinical endpoints (101/173, 58.38%) (*P<*0.001). Compared to single-center trials (327/479, 68.27%) or those with an unknown study site (33/49, 67.35%), multicenter trials (266/328, 81.10%) were more likely to report results (*P<*0.001). Furthermore, trials with longer duration had a higher frequency of disclosed results (*P<*0.001). There were no significant differences in the availability of trial results in terms of the completion date before or after 1 January 2008, source of funding, the country of trial origin, and reasons for trial termination or suspension.

**Table 1:** Characteristics of included clinical trials investigating PC.

Total trials (n=856)	Results available (n=626)		Results not available (n=230)	*P*-value
Before 1 January 2008	219 (34.98)		89 (38.70)	0.316
After 1 January 2008	407 (65.02)		141 (61.30)	
Primary endpoint met (%)	No (n=85)	Yes (n=541)		
1. Registered on clinicaltrials.gov				
a. Yes	36 (42.35)	213 (39.37)		0.602
b. No	49 (57.65)	328 (60.63)		
2. Published results				
a. Yes	55 (64.71)	484 (89.46)		**< 0.001**
b. No	30 (35.29)	57 (10.54)		
Study type				
1. Interventional	578 (92.33)		179 (78.11)	**< 0.001**
2. Observational	48 (7.67)		51 (22.17)	
Study status				
1. Completed	514 (82.11)		154 (66.96)	**< 0.001**
2. Terminated or suspended	112 (17.89)		76 (33.04)	
Intervention				
1. Procedure	52 (8.31)		36 (15.65)	**< 0.001**
2. Device	23 (3.67)		10 (4.35)	
3. Drug	415 (66.29)		109 (47.39)	
4. Radiation	23 (3.67)		9 (3.91)	
5. Biological	64 (10.22)		20 (8.70)	
6. Other	39 (6.23)		30 (13.04)	
7. Unknow	10 (1.60)		16 (6.96)	
Phase				
1. I	104 (16.61)		57 (24.78)	**< 0.001**
2. II	340 (54.31)		70 (30.43)	
3. III	52 (8.31)		17 (7.39)	
4. IV	4 (0.64)		4 (1.74)	
5. Unknow/not available	126 (20.13)		82 (35.65)	
Funding source				
1. NIH or Federal	21 (3.35)		6 (2.61)	0.376
2. Industry	150 (23.96)		46 (20.00)	
3. Other	455 (72.68)		178 (77.39)	
Endpoint				
1. Clinical	525 (83.86)		158 (68.70)	**< 0.001**
2. Non-clinical	101 (16.13)		72 (31.30)	
Centers				
1. Single	327 (52.24)		152 (66.09)	**< 0.001**
2. Multiple	266 (42.49)		62 (26.96)	
3. Unknow	33 (5.27)		16 (6.96)	
Country of trial origin				
1. North America	357 (57.03)		135 (58.70)	0.616
2. Europe	118 (18.85)		42 (18.26)	
3. Asia	59 (9.42)		23 (10.00)	
4. Multiple countries	54 (8,63)		12 (5.22)	
5. Other	5 (0.80)		2 (0.87)	
6. Unknown	33 (5.27)		16 (6.96)	
Median trial duration in weeks (IQR)	3.75 (2.41, 5.42)		3.00 (1.92, 4.70)	**< 0.001**
Reason for termination or suspension of trial (n=188)				
1. Enrolment issues	10 (8.93)		4 (5.26)	0.700
2. Safety concerns, adverse events, interim analysis	16 (14.29)		8 (10.53)	
3. Medical futility or lack of efficacy	19 (16.96)		11 (14.47)	
4. Issues related to funding, personnel, supplies, local or federal regulation	35 (31.25)		28 (36.84)	
5. Unclear	32 (28.57)		25 (32.89)	

Bold text indicated the statistical difference of the P value between groups.

### 3.3 Contributing factors of results availability

**Table 2** shows the results of the logistic regression analysis. Compared to observational trials, interventional trials had better availability of the results (OR=2.591, 95% CI 1.297-5.180, *P*=0.007). Completed trials had higher availability of results than terminated or suspended trials (OR=2.624, 95% CI 1.796-3.834, *P*<0.001). Drug-related trials were more likely to disclose the results of trials than trials with unknown interventions (OR=3.758, 95%CI: 1.299-10.980, *P*=0.015). Phase II trials (OR=2.512, 95% CI 1.630-3.873, *P*<0.001) and trials with unknown or not available Phase details (OR=2.982, 95% CI 1.465-6.069, P=0.003) had better results availability than phase I trials. Furthermore, the duration of the trial in years was found to be a contributing factor in the availability of the results (OR=1.070, 95%CI: 1.000-1.145, *P*=0.050).

**Table 2:** Multivariate logistic regression for influencing factors of the availability of PC clinical trial results.

Trial characteristics	OR (95% CI)	*P* value
Interventional vs. observational	2.591 (1.297, 5,175)	**0.007**
Completed vs. terminated or suspended	2.624 (1.796, 3.834)	**<0.001**
Intervention		
1. Procedure	1.534 (0.564, 4.175)	0.402
2. Device vs. unknown	1.691 (0.484, 5.902)	0.410
3. Drug vs. unknown	3.758 (1.299, 10.980)	**0.015**
4. Radiation vs. unknown	2.763 (0.771, 9.905)	0.119
5. Biological treatment vs. unknown	2.780 (0.863, 8.956)	0.087
6. Other treatment vs. unknown	1.486 (0.550, 4.012)	0.435
Phase		
1. II vs. I	2.512 (1.630, 3.873)	**<0.001**
2. III vs. I	1.594 (0.804, 3.161)	0.182
3. IV vs. I	0.579 (0.130, 2.587)	0.475
4. Unknown/not available vs. phase I	2.982 (1.465, 6.069)	**0.003**
Clinical vs. Non-clinical	1.318 (0.835, 2.081)	0.235
Centers		
1. Multiple vs. single	1.437 (0.981, 2.103)	0.062
2. Unknow vs. single	0.902 (0.452, 1.802)	0.771
Median trial duration in years (IQR)	1.070 (1.000, 1.145)	**0.050**

Bold text indicated the statistical difference of the P value between groups.

## 4 Discussion

Public disclosure of clinical trial results is important both for clinical decision-making and for dissemination of knowledge (17). However, due to the complexity and unknown outcomes of clinical trials and the inattention of some study coordinators, the current availability of clinical trial results is not satisfactory in many fields (18). To identify the disclosure rate of clinical trial results in PC, we conducted this study and analyzed relevant factors that may affect the availability of clinical trial results. Our study provided preliminary evidence for the transparency of PC clinical trials and provided a reference for the design and development of PC clinical trials in the future.

In terms of clinical trials with available results, we found that 86.10% (539/626) eventually reported their findings in publications, but only 39.78% (249/626) reported the results on ClinicalTrials.gov. While a publication rate of approximately 90% was satisfactory, a 40% reporting rate of results on ClinicalTrials.gov suggested that most researchers seemed to have ignored the significance of ClinicalTrials.gov for information disclosure of clinical trial results and there was still room for improvement in the disclosure of PC clinical trial results. Given the current open-access policy for journals, ClinicalTrials.gov remained a more rapid, efficient, and less expensive option for accessing trial results. Therefore, we suggested that sponsors of clinical trials, publishers and editors of journals should strengthen their review of ClinicalTrials.gov during the development of clinical trials and on the submission of articles for publication to ensure that all clinical trial results were disclosed on ClinicalTrials.gov. In addition, we found that 26.87% of the trials did not disclose any information about the results. Similar results have been found in previous studies. In one study evaluating the transparency of clinical trials in ovarian cancer, about 25% of the results of clinical trials were not published in any form (19). Nguyen et al. found that approximately half of US cancer drug trials were not published three years after the trials were completed (20). This indicated that, in addition to PC, clinical trials in other cancer fields also have some problems with transparency. Owing to the highly malignant nature and the extremely low long-term survival rate of PC, timely disclosure of the results of clinical trials is of great importance. On the one hand, trials that meet the primary endpoint can help clinicians better formulate the treatment of patients to delay disease progression and improve prognosis; on the other hand, failed trial results or adverse reactions in trials can prevent the continuation of such studies and harm to patients in treatment. Therefore, we believe that irrespective of the results of clinical trials, researchers should comply with the FDAAA and establish knowledge sharing and report the content and results of the trial in a timely manner to improve the transparency of data derived from clinical trials.

In this study, we also identified characteristics of clinical trials that were associated with the availability of results, such as interventional trials, completed trials, drug-related trials, phase II trials, and trials of longer duration, which may provide an important reference for researchers when designing trial protocols. We believed that compared with observational studies, the process of recruitment and ethical review of interventional studies are more complex and stricter, and the disclosure requirements for them are also higher, which may be one of the reasons for the high availability of results. Compared to completed studies, we found that more terminated or suspended studies failed to disclose information about trial results on ClinicalTrials.gov (40.43% VS. 23.05%). Researchers seemed to neglect the disclosure of trial information because of the interruption of the study process. In addition, drug-related interventions were found to be significantly correlated with the availability of trial results in our study. Considering the high malignancy of PC, patients still have a high risk of recurrence and a poor prognosis even after radical resection. At present, chemotherapy protocols based on fluorouracil have become the standard treatment strategy for PC worldwide (21). To improve the disease condition and prognosis of patients, many large-scale clinical trials of perioperative and advanced first-line chemotherapy for PC patients are being carried out (22). Therefore, drug-related clinical trials had a higher disclosure rate regardless of trial results. Although the introduction of the FDAAA in 2007 did not have a significant impact on the availability of results in this study, it was undeniable that a higher proportion of trials registered after 1 January 2008 disclosed their study results (65.02% vs. 61.30%). It can be seen that the FDAAA did have a certain positive significance for improving the transparency of clinical trials. Additionally, 40.43% (76/188) of the terminated or suspended trials did not report any results. In future, researchers should pay closer attention to the reasons for changes in the progression of the trials and report their findings in a timely manner to avoid unnecessary waste of resources and adverse effects on patients.

This study also has certain limitations. We only included PC-related clinical trials registered on ClinicalTrials.gov for analysis, and clinical trial registration platforms in other countries or not in English were not included in our study, which may lead to certain selection bias. Based on our study, future studies should expand eligibility criteria and include more clinical trial registration platforms to enrich findings.

## 5 Conclusions

This study was the first to examine the transparency in the divulgation of data obtained from clinical trials involving patients with PC. Approximately, 26.87% of clinical trials did not disclose any information about their results and of the trials with available results, 60.22% did not disclose findings on ClinicalTrials.gov. Overall, there is still room for improving the transparency data deriving from clinical trials involving patients with PC.

## Data availability statement

The raw data supporting the conclusions of this article will be made available by the authors, without undue reservation.

## Author contributions

L-HH was the principal investigator for this project and made critical revisions to the article. R-QH, YZ and H-XZ were responsible for analyzing and interpreting the data and drafting the manuscript. DW and X-YZ assisted in data collection, analysis. Z-SL and L-HH supervised the research process and provided technical suggestions. All authors contributed to the article and approved the submitted version.
