# Supplementary material for: Transparency of clinical trials in pancreatic cancer: An analysis of availability of trial results from the ClinicalTrials.gov database
Source: Front Oncol. 2023 Jan 6;12:1026268. doi: 10.3389/fonc.2022.1026268 (PMC9853913; doi:10.3389/fonc.2022.1026268)
Supplement: Supplementary file 1 [file DataSheet_1.docx]

Supplementary Material

# Definitions

Trial results were considered available when provided on ClinicalTrials.gov or in a published format. Publication was defined as the first abstract or article published in a peer-reviewed journal that was 1) consistent with the study’s title and aim(s) and 2) conducted by the listed investigator(s) on the listed trial site(s).

Observational trials were defined by ClinicalTrials.gov as studies in which participants have been identified as part of study groups and have undergone a biomedical or health outcome evaluation.

Interventional trials were defined by ClinicalTrials.gov as studies in which participants have been assigned to groups receiving or not receiving one or more interventions. Interventions were classified into Procedure, Device, Drug, Radiation, Biological, other, and unknown based on the data obtained from ClinicalTrials.gov. All other interventions were grouped as the other. Trials without any listed intervention on ClinicalTrials.gov were classified as unknown.

For study status, suspended was defined by ClinicalTrials.gov as studies have stopped early but may start again; terminated was defined by ClinicalTrials.gov as studies have stopped early and will not start again, and participants would no longer be examined or treated; completed was defined by ClinicalTrials.gov as studies have ended normally, and participants would no longer be examined or treated (that is, the last participant's last visit has occurred).

Trial stage was derived from ClinicalTrials.gov. Phases were classified as: Phase Ⅰ, Phase Ⅱ, Phase Ⅲ and Phase Ⅳ.

Phase Ⅰ, a phase of research to describe clinical trials that focus on the safety of a drug, aims to determine the drug's most frequent and serious adverse events and, often, how the drug is broken down and excreted by the body.

Phase Ⅱ, a phase of research to describe clinical trials that gather preliminary data on whether a drug works in people who have a certain condition/disease (that is, the drug's effectiveness).

Phase Ⅲ, a phase of research to describe clinical trials that gather more information about a drug's safety and effectiveness by studying different populations and different dosages and by using the drug in combination with other drugs.

Phase Ⅳ, a phase of research to describe clinical trials occurring after the FDA has approved a drug for marketing. These trials gather additional information about a drug's safety, efficacy, or optimal use.

Phase that not listed or not applicable (e.g., for observational trials) was categorized as unknown/not applicable.

Sources of funding were categorized into 1) NIH or the federal government (trial was partially or fully funded by NIH or the United States), 2) industry (trial was partially or fully funded by federal subsidization or industry), 3) other (trial was partially or fully funded by individuals, universities and community organizations).

Clinical endpoints were defined as outcomes directly related to patients (e.g. mortality, hospital-admission, adverse events etc.).

Non-clinical endpoints were defined as all other outcomes (e.g. change in laboratory or endoscopic measures).

Country of trial origin was determined by study location(s) reported on ClinicalTrials.gov., and was categorized into North America, Europe, Asia, multiple countries (for trial locations in more than one country), other (South and Central America, Middle East, Australia, New Zealand and Africa), and unknown (trials without any listed location(s)).

Reason for termination data was abstracted from ClinicalTrials.gov. and was classified into 1) Enrolment issues, 2) Safety concern, adverse events, interim analysis, 3) Medical futility or lack of efficacy, 4) Issues related to funding, personnel, supplies, local or federal regulation, and 5) unclear (trials without unclear reasons for termination).
